# Association of PFAS, Metals, Phthalate and Organophosphate Metabolites with Depression Among U.S. Adults

**DOI:** 10.3390/ijerph23020205

**Published:** 2026-02-06

**Authors:** Olamide Ogundare, Emmanuel Obeng-Gyasi

**Affiliations:** 1 Department of Built Environment, North Carolina A&T State University, Greensboro, NC 27411, USA; 2 Environmental Health and Disease Laboratory, North Carolina A&T State University, Greensboro, NC 27411, USA

**Keywords:** depression, organophosphate metabolites, phthalates, PFAS, heavy metals, multi-exposure

## Abstract

**Highlights:**

**Public health relevance—How does this work relate to a public health issue?**

- Depression is a major public health concern, and this study evaluates whether exposure to common environmental chemicals is associated with depressive symptoms in the U.S. population.
- The analysis examines PFAS, metals, phthalates, and organophosphate metabolites that are widely present in food, consumer products, and indoor environments.

**Public health significance—Why is this work of significance to public health?**

- Specific organophosphate and phthalate metabolites were associated with higher PHQ-9 scores, indicating potential environmental contributions to depressive symptoms.
- Mixture modeling showed that organophosphate metabolites had the strongest overall contribution, highlighting the importance of evaluating combined chemical exposures rather than individual compounds alone.

**Public health implications—What are the key implications or messages for practitioners, policy makers and/or researchers in public health?**

- Environmental exposures should be considered in public health research and surveillance related to mental health outcomes.
- The findings support efforts to reduce population-level exposure to neurotoxic and endocrine-disrupting chemicals and highlight the need for longitudinal studies to clarify temporal relationships.

**Abstract:**

Depression is a major public health concern, and evidence continues to show that environmental toxicants may contribute to its development. This study evaluated the association between depressive symptoms and per- and polyfluoroalkyl substances (PFAS), heavy metals, phthalates, and organophosphate metabolites using data from NHANES 2017–2018. Depressive symptoms were measured with the Patient Health Questionnaire-9 (PHQ-9). Environmental exposure variables were analyzed using multivariable linear regression and Bayesian Kernel Machine Regression (BKMR). All models adjusted for demographic, socioeconomic, behavioral, and clinical covariates. In multivariable linear regression models adjusted for demographic, socioeconomic, behavioral, and clinical covariates, higher urinary dimethylphosphate concentrations were significantly associated with increased depressive symptom scores (β = 0.15; 95% CI: 0.04, 0.27; *p* = 0.0098). Mono-(2-ethylhexyl) phthalate (MEHP) was also positively associated with PHQ-9 scores (β = 0.001; 95% CI: 0.0003, 0.0019; *p* = 0.0043). Because environmental mixtures tend to follow non-linear patterns, BKMR analysis was run. BKMR analyses indicated that organophosphate metabolites exhibited the greatest overall contribution to depressive symptoms (group posterior inclusion probability = 0.7875), with diethylphosphate emerging as the most influential individual exposure within the group (conditional PIP = 0.7211). Exposure–response functions suggested non-linear and threshold relationships for several metabolites. These findings identify specific organophosphate and phthalate metabolites as potential contributors to depressive symptoms and support the importance of evaluating chemical mixtures rather than single exposures. Additional longitudinal studies are needed to clarify temporal relationships and to inform public health efforts aimed at reducing exposure to organophosphate pesticides and endocrine-disrupting chemicals.

## 1. Introduction

Depression remains one of the foremost contributors to the global burden of mental health disorders, impacting an estimated 300 million individuals across the world [1]. Depressive symptoms, a highly prevalent mental health condition, are characterized by a persistent depressed mood, markedly diminished interest in routine activities, and a reduced capacity to experience pleasure. Emerging research increasingly links these symptoms to environmental toxicants, such as per- and polyfluoroalkyl substances (PFAS), heavy metals (e.g., lead, cadmium, mercury), and endocrine-disrupting compounds [2,3,4]. Among the endocrine-disrupting compounds, there is growing evidence that urinary biomarkers of phthalates and organophosphates, two classes of environmental toxicants, are independently associated with increased risk of depression in adults. These exposures can disrupt endocrine and neurological pathways, possibly influencing mood regulation, stress response, and neurotransmitter systems [5,6].

Lead, cadmium, and mercury are well-established neurotoxic metals with biological pathways that plausibly link them to depressive symptoms [2,3,5]. These metals can induce oxidative stress, disrupt mitochondrial function, and promote neuroinflammation, processes that impair neuronal signaling and have been implicated in mood dysregulation. Lead exposure has been associated with altered neurotransmitter activity, particularly within dopaminergic and serotonergic pathways [3,4]. Cadmium can interfere with calcium-dependent neuronal processes and has been linked to increased inflammatory cytokine production [4]. Mercury, especially in its organic forms, can cross the blood–brain barrier and disrupt neuronal membrane integrity and synaptic function [4].

Phthalates are a large family of synthetic chemicals widely used as plasticizers (substances added to plastics to increase their flexibility, transparency, durability, and longevity). They are commonly found in personal care products (shampoos, lotions, perfumes), food packaging and plastic containers, toys, vinyl flooring, and medical devices (IV tubing, blood bags) [7]. Phthalates are classified into two main subgroups based on their molecular weight: low-molecular-weight phthalates and high-molecular-weight phthalates. The low-molecular-weight phthalates constitute a distinct subgroup within the phthalate family, characterized by shorter alkyl side chains (3–6 carbon atoms) and reduced molecular mass. Owing to these chemical attributes, they are predominantly utilized in non-polyvinylchloride (PVC) applications, including cosmetics, personal care formulations, and various household products. On the other hand, high-molecular-weight (HMW) phthalates are phthalates with longer, heavier alkyl side chains (≥7 carbon atoms). Because of their physical properties, they are widely used as plasticizers in polyvinyl chloride (PVC) to make plastics flexible, durable, and long-lasting [8,9].

A 2015 study using NHANES data found that higher urinary concentrations of specific phthalate metabolites, such as mono-n-butyl, mono-isobutyl, and mono-benzyl phthalates were significantly associated with adult depression, even after adjusting for health history and lifestyle factors [10]. Phthalates are known endocrine disruptors which have been linked to a range of health effects. In the male reproductive development, exposure during pregnancy has been associated with reduced anogenital distance AGD (the distance between the anus and the base of the penis) in male infants and increased risk of undescended testes, which may lead to infertility and testicular cancer [11,12]. Female reproductive health studies suggest potential impacts on ovarian function and menstrual cycle regulation [13]. In the developmental and neurobehavioral effects, prenatal exposure may affect neurodevelopment, with some studies linking phthalates to behavioral issues and lower IQ in children [14,15]. Phthalate exposure has been associated with metabolic and hepatic dysfunction, including insulin resistance, obesity, and liver toxicity [16,17], biological processes that are increasingly recognized as upstream contributors to neuroinflammation, hypothalamic–pituitary–adrenal axis dysregulation, and depressive symptomatology. In addition, phthalates can cross the placental barrier and induce long-lasting epigenetic modifications that alter gene expression [18,19], potentially influencing neurodevelopmental and neuroendocrine pathways implicated in mood regulation.

Organophosphates are a class of synthetic compounds used as insecticides/pesticides in agriculture, public health and sometimes in households. Organophosphate pesticides once absorbed into the human body primarily through inhalation, ingestion, or dermal exposure undergo enzymatic biotransformation in the liver. These compounds are metabolized into water-soluble dialkyl phosphate (DAP) metabolites, which are subsequently excreted in urine. Common DAPs include Dimethylphosphate (DMP), Diethylphosphate (DEP), Dimethylthiophosphate (DMTP), and Diethylthiophosphate (DETP) [20,21,22]. These metabolites serve as biomarkers of exposure, reflecting recent contact with a wide range of organophosphorus pesticides, though they do not indicate the specific parent compound. Phthalates are semivolatile organic compounds (SVOCs) frequently detected in indoor environments, while organophosphate pesticides are primarily encountered through agricultural and residential pesticide use. Although they differ in their applications, both can contribute to human exposure through overlapping pathways such as dietary intake, indoor contamination following residential pesticide use, and contact with contaminated dust or surfaces. Phthalates function primarily as plasticizers in materials such as vinyl flooring, personal care products, and food packaging, whereas organophosphate pesticides (distinct from organophosphate ester flame retardants) are primarily encountered through agricultural and residential pesticide use, including dietary intake and household pesticide applications. Phthalates readily accumulate in household dust and indoor environments, including heating, ventilation, and air conditioning (HVAC) systems and filters, whereas exposure to organophosphate pesticides primarily occurs through dietary intake and residential pesticide application. While phthalates are commonly encountered via inhalation, dermal contact, and indoor dust, exposure to organophosphate pesticides primarily occurs through dietary intake and residential pesticide application [23,24].

High levels of organophosphate metabolites have been linked to neurological effects, especially in children and vulnerable populations. Chronic exposure in children may contribute to developmental delays, cognitive impairment, and hormonal disruption [25,26]. Organophosphates are also linked to mood disorders (e.g., depression, anxiety), mood instability, including episodes of tearful depression, irritability, and impulsive suicidal ideation. A case study by Davies et al. described a farmer with long-term organophosphate exposure who developed severe mood swings, apathy, and suicidal thoughts, highlighting the psychiatric toll of chronic exposure [27]. These compounds (Phthalates and Organophosphate) are often found together in the same microenvironments (e.g., homes, schools, offices), hence understanding their combined exposure is critical for accurate risk assessment.

Extensive research has examined the link between PFAS exposure and depressive symptoms in adults, with growing attention to its impact on maternal health outcome [28,29]. A review investigating shared metabolic disturbances in PFAS-exposed pregnant individuals and those experiencing perinatal or antenatal depression uncovered potential biochemical pathways that may underline this connection. Simultaneously, environmental exposure to toxic metals such as lead, cadmium, and mercury has been independently correlated with negative impacts on mental health, including disruptions in cognitive performance and emotional well-being [30].

Although heavy metals, organophosphates, and phthalates have each been independently linked to neurotoxicity and depressive symptoms, real-world exposures occur as complex chemical mixtures rather than isolated compounds. Evidence on the joint and interactive effects of these chemical classes remains limited, despite overlapping biological pathways and partially overlapping exposure contexts, including oxidative stress, endocrine disruption, neuroinflammation, and dysregulation of stress-response systems. Consequently, traditional single-chemical approaches may underestimate the combined neurotoxic burden of co-occurring environmental exposures. Advanced mixture modeling approaches are therefore needed to better characterize these combined effects and their relevance to mental health outcomes. The objective of this study is to examine the association between depressive symptoms and the combined influence of environmental exposures.

## 2. Materials and Methods

### 2.1. Study Design

This retrospective cross-sectional study utilizes data from the 2017–2018 cycle of the National Health and Nutrition Examination Survey (NHANES), a survey of the United States population conducted by the Centers for Disease Control and Prevention (CDC). This includes a sample of U.S. adults. Participants were eligible for inclusion if they had complete data on depressive symptoms, PFAS biomarkers, metal biomarkers, phthalate metabolites, and organophosphate metabolites. Individuals were excluded if they were pregnant, had missing exposure or outcome data, or lacked key sociodemographic covariates required for analysis. After applying these criteria, a total sample of 179 participants was included in the analytic dataset.

The analysis examines the relationship between PFAS, metals, phthalate and organophosphate metabolites, and depressive symptoms in a U.S. sample. The analyses adjusted for factors such as age, sex, BMI, ethnicity and socioeconomic status (income and education), providing a nuanced understanding of population-level health trends. NHANES employs a multi-stage, stratified sampling design and collects data biennially through physical examinations, laboratory testing, and structured interviews. Demographic information is gathered using a Computer-Assisted Personal Interview (CAPI) system to ensure accuracy. All protocols were reviewed and approved by the Institutional Review Board at the National Center for Health Statistics (NCHS), a division of the CDC.

The National Health and Nutrition Examination Survey (NHANES) protocols were conducted in accordance with the ethical principles of the Declaration of Helsinki. Written informed consent was obtained from all participants prior to data collection. The NHANES 2017–2018 survey cycle was reviewed and approved by the Ethics Review Board of the National Center for Health Statistics (NCHS), Centers for Disease Control and Prevention, under Protocol #2018-01, which became effective on 26 October 2017, and represents a continuation of Protocol #2011-17. The present study in-volved a secondary analysis of de-identified, publicly available NHANES data and did not require additional institutional review or trial registration. The present analysis involved secondary analysis of de-identified, publicly available data and did not require additional institutional review or trial registration.

#### Questionnaire Administration and Sample Collection

Questionnaire data were collected through standardized NHANES interviews conducted by trained personnel using a computer-assisted personal interviewing (CAPI) system. Participants completed structured questionnaires that captured sociodemographic characteristics, health behaviors, medical history, and mental health information, including depressive symptoms. Interviews were conducted in participants’ homes or in the Mobile Examination Center (MEC), following uniform protocols to ensure consistency and data quality. Biological samples were collected during the MEC examination. Blood specimens were obtained by certified phlebotomists using standardized venipuncture procedures, while urine samples were self-collected under supervision using sterile col lection materials. All specimens were processed, aliquoted, and stored under controlled conditions before being shipped to NHANES contract laboratories for chemical analysis. Sample handling, storage, and transport followed strict quality-control procedures to maintain specimen integrity and ensure reliable quantification of environmental toxicants.

### 2.2. Exposure Assessment

Biomarkers of per- and polyfluoroalkyl substances (PFAS), heavy metals, phthalate metabolites, and organophosphate metabolites were quantified by the National Center for Environmental Health (NCEH), Centers for Disease Control and Prevention (CDC), using standardized laboratory protocols implemented in the NHANES 2017–2018 cycle. All laboratory analyses were conducted at the CDC Division of Laboratory Sciences (Atlanta, GA, USA). Across all analyte classes, biological specimens were collected, processed, stored, and analyzed according to NHANES laboratory protocols. These procedures included standardized sample storage conditions, use of isotope-labeled internal standards, calibration with certified reference materials, and routine quality assurance and quality control measures such as reagent blanks, duplicate samples, and periodic reanalysis to ensure analytical precision and accuracy.

#### 2.2.1. PFAS Measurement

Serum concentrations of PFAS, including perfluorooctanoic acid (PFOA) and perfluorooctane sulfonic acid (PFOS), were measured using solid-phase extraction followed by high-performance liquid chromatography with isotope dilution tandem mass spectrometry (HPLC–MS/MS). Analyses were performed using an Agilent 1200 HPLC system (Agilent Technologies, Santa Clara, CA, USA) coupled with an AB Sciex API 5500 triple quadrupole mass spectrometer (AB Sciex, Framingham, MA, USA). Isotope-labeled internal standards (Wellington Laboratories, Guelph, ON, Canada) were added to all samples prior to extraction to correct for matrix effects and analytical variability. Calibration curves were generated using certified reference materials, and method detection limits were in the sub–ng/mL range. Serum specimens were stored at −20 °C prior to analysis.

#### 2.2.2. Measurement of Heavy Metals

Whole-blood concentrations of lead, cadmium, and mercury were quantified using inductively coupled plasma mass spectrometry (ICP-MS). Analyses were conducted using a PerkinElmer NexION 300D ICP-MS instrument (PerkinElmer, Waltham, MA, USA). Blood samples were diluted with a matrix-modifying solution containing ammonium hydroxide, ethylenediaminetetraacetic acid (EDTA), Triton X-100 (Sigma-Aldrich, St. Louis, MO, USA), and ultrapure deionized water.

Indium and rhodium were used as internal standards to correct for instrumental drift, and calibration was performed using National Institute of Standards and Technology (NIST) traceable reference materials. Whole blood specimens were stored under refrigerated or frozen conditions in accordance with NHANES protocols prior to analysis.

#### 2.2.3. Phthalate Metabolite Measurement

Urinary phthalate metabolites were measured using high-performance liquid chromatography coupled with electrospray ionization tandem mass spectrometry (HPLC-ESI-MS/MS). Analyses were performed on a Waters Acquity HPLC system (Waters Corporation, Milford, MA, USA) interfaced with an AB Sciex API 4000 mass spectrometer (Framingham, MA, USA). Urine samples underwent enzymatic deconjugation using β-glucuronidase (Roche Diagnostics, Indianapolis, IN, USA), followed by online solid-phase extraction. Stable isotope-labeled internal standards were added prior to sample preparation. Quantified metabolites included mono-ethyl phthalate (MEP), mono-n-butyl phthalate (MBP), mono-(2-ethylhexyl) phthalate (MEHP), mono-(2-ethyl-5-hydroxyhexyl) phthalate (MEHHP), and mono-(2-ethyl-5-carboxypentyl) phthalate (MECPP). Method detection limits were generally in the low ng/mL range. Urine specimens were stored at −20 °C prior to analysis.

#### 2.2.4. Organophosphate Metabolite Measurement

Urinary organophosphate metabolites, including dimethylphosphate (DMP), diethylphosphate (DEP), and dimethylthiophosphate (DMTP), were quantified following CDC laboratory protocols. Urine samples were collected in sterile polypropylene containers and stored at 4 °C during transport before being frozen until analysis at the CDC laboratory. Samples underwent enzymatic hydrolysis to cleave conjugated metabolites, followed by solid-phase extraction using Oasis HLB cartridges (Waters Corporation, Milford, MA, USA). Extracts were analyzed using ultrahigh-performance liquid chromatography coupled with triple quadrupole tandem mass spectrometry (UHPLC–MS/MS) on a Thermo Scientific Dionex Ultimate 3000 UHPLC system paired with a TSQ Vantage mass spectrometer (Thermo Fisher Scientific, Waltham, MA, USA). Isotope-labeled internal standards were used for quantification. Quality assurance procedures included reagent blanks, blind split samples, and repeat analysis of approximately 2% of specimens.

### 2.3. Outcome Assessment: Depression

Depression was evaluated using the Patient Health Questionnaire-9 (PHQ-9) [31], administered during in-person interviews at NHANES mobile examination centers (MEC). Participants reported the frequency of nine depressive symptoms experienced over the preceding two weeks, rated on a scale from 0 (not at all) to 3 (nearly every day). These symptoms included anhedonia, persistent sadness, sleep disturbances, fatigue, appetite changes, feelings of worthlessness, difficulty concentrating, psychomotor changes, and thoughts of self-harm. The total PHQ-9 score ranges from 0 to 27, with scores ≥10 indicating clinically significant depression. The PHQ-9 is a widely validated instrument with strong internal consistency, sensitivity, and specificity for identifying major depressive disorders. Severity categories are defined as minimal (1–4), mild (5–9), moderate (10–14), moderately severe (15–19), and severe (20–27).

### 2.4. Statistical Analysis

#### 2.4.1. Descriptive Statistics

Descriptive statistics were used to characterize the study population and exposure distributions. Demographic variables including age, sex, race/ethnicity, education level, and income were summarized using means and standard deviations for continuous variables, and frequencies and percentages for categorical variables. Lifestyle factors such as smoking status, alcohol consumption, and physical activity were also described. Urinary concentrations of phthalate and organophosphate metabolites were reported as medians and interquartile ranges (IQRs). Depression was defined using the Patient Health Questionnaire (PHQ-9), with a score ≥ 10 indicating moderate to severe depressive symptoms. Prevalence of depression was stratified across quartiles of exposure to assess initial trends.

#### 2.4.2. Linear Regression

Multivariable linear regression model was used to evaluate the relationship between urinary concentrations of phthalate and organophosphate metabolites and depressive symptoms. The primary outcome was the continuous Patient Health Questionnaire-9 (PHQ-9) score, which quantifies the severity of depressive symptoms. Models adjusted for key confounders including age, sex, socioeconomic status, body mass index, smoking status, and urinary creatinine to account for dilution. Metabolite concentrations modeled both continuously and by quartiles to assess dose response.

#### 2.4.3. Handling of Missing Data

Analyses were restricted to participants with complete data on all exposure variables, covariates, and outcomes of interest. Participants with missing values were excluded from the analytic sample. This complete-case approach was adopted to ensure consistency across linear regression and Bayesian kernel machine regression analyses.

#### 2.4.4. Bayesian Kernel Machine Regression (BKMR)

Bayesian Kernel Machine Regression (BKMR) was used to evaluate the joint, non-linear, and potentially interactive effects of correlated urinary phthalate and organophosphate metabolites on depression. This approach is well-suited for environmental mixture analysis, allowing flexible modeling of complex exposure–response relationships while adjusting for confounding. All measured urinary metabolites were included as a multivariate exposure matrix, grouped into two chemical classes: phthalates and organophosphates. Depression status served as the primary outcome, modeled as a continuous variable. Covariates were included as linear predictors and matched those used in the linear regression models. In this analysis, age, sex, race/ethnicity, body mass index (BMI), smoking status, alcohol use, and income were adjusted for.

BKMR was implemented using the BKMR package in R (version 4.5.1; R Foundation for Statistical Computing, Vienna, Austria), with 50,000 iterations. The model estimated the overall effect of the exposure mixture, identified individual contributors, and visualized exposure-response relationships and interactions. To assess variable importance, group posterior inclusion probabilities (group PIPs) were calculated to evaluate whether entire chemical classes (phthalates or organophosphates) were jointly associated with depression. Conditional PIPs were also examined which estimate the importance of each individual metabolite conditional on the presence of others in the model. These metrics provided insight into both the collective and specific contributions of urinary biomarkers to depression risk.

## 3. Results

### 3.1. Descriptive Statistics Results

Table 1a,b presents descriptive statistics for the continuous variables (N = 179). Participants were middle-aged (mean age = 44.39 years) with an average BMI of 29.57, indicating an overweight-to-obese cohort. The mean PHQ-9 score (4.84) reflected mild depressive symptoms. PFOS concentrations (5.64 ng/mL) exceeded PFOA (1.60 ng/mL), suggesting differing exposure sources. Heavy metal levels were low overall, though mercury showed greater variability. Organophosphate metabolites, including dimethylphosphate (mean = 3.00 µg/L, SD = 7.70) and diethylphosphate (mean = 4.39 µg/L, SD = 6.36), exhibited moderate means but substantial variability, suggesting heterogeneous pesticide exposure among participants. Similarly, phthalate metabolites varied widely, with MEHP showing a standard deviation (SD) of 970.81. The participant sample in this study as seen in Table 1b comprised 109 males (60.89%) and 70 females (39.11%), with non-Hispanic individuals representing the largest ethnic group at 44.69% of the study population

### 3.2. Linear Regression

Table 2 displays the linear regression results with regression coefficients, standard errors, *p*-values, and 95% confidence intervals for the associations between environmental exposures and depressive symptoms. Most environmental chemicals, including PFOA, PFOS, lead, cadmium, mercury, and most phthalate metabolites show non-significant associations with depressive symptoms, as indicated by *p*-values greater than 0.05 and confidence intervals that cross zero. These findings suggest no meaningful linear relationship between these exposures and PHQ scores in this model.

Among the organophosphate metabolites, Dimethylphosphate demonstrates a statistically significant positive association with depressive symptoms (β = 0.154, *p* = 0.0098), with a confidence interval (0.0375, 0.2695) that excludes zero. This suggests that higher Dimethylphosphate levels are associated with increased PHQ scores. Diethylphosphate shows a borderline association (β = 0.126, *p* = 0.0648), indicating a possible but not definitive relationship. Dimethylthiophosphate, however, does not show evidence of an association. MEHP also exhibits a statistically significant positive association (β = 0.001, *p* = 0.0043), with a very narrow confidence interval that does not include zero. Although the effect size is small, the direction and statistical significance indicate that higher MEHP levels may be linked to increased depressive symptoms.

Overall, the results suggest that most chemical exposures in the model do not have strong linear associations with depressive symptoms. However, two exposures, Dimethylphosphate and MEHP, emerge as significant predictors, indicating that specific organophosphate and phthalate metabolites may play a role in depression-related outcomes.

### 3.3. Spearman Correlation Analysis

**Figure 1:**
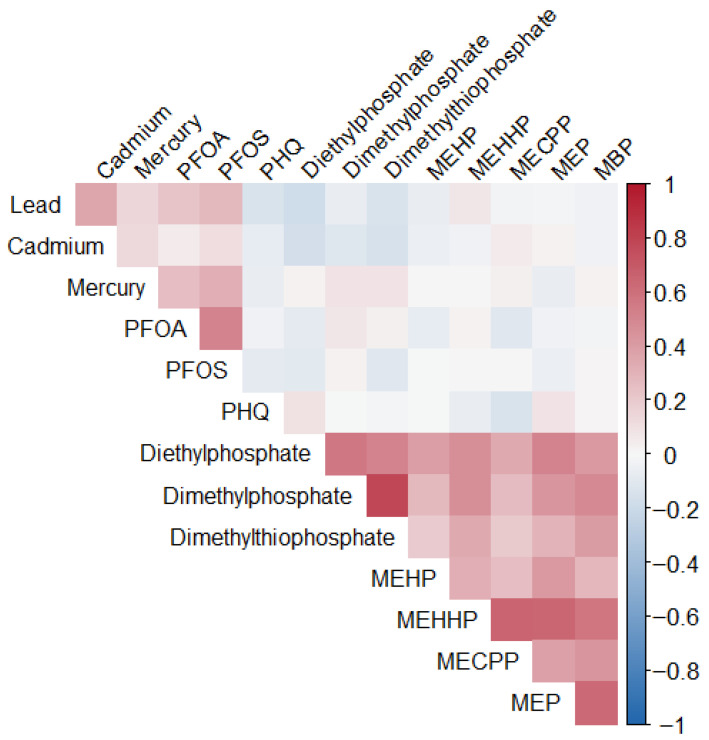
Spearman Correlation among variables of interest.

Figure 1 displays the Spearman correlation matrix for the study variables, including metals, PFAS, organophosphate metabolites, phthalates, and PHQ scores. Several notable positive correlations are observed. PFOA and PFOS show a moderate correlation (0.52), reflecting shared exposure sources. Lead correlates moderately with cadmium (0.36) and weakly with mercury (0.15), PFOA (0.22), and PFOS (0.28). Mercury also shows moderate correlations with PFOS (0.32) and PFOA (0.25).

The strongest correlations appear among organophosphate metabolites, particularly between Dimethylphosphate and Dimethylthiophosphate (0.79), indicating common pesticide exposure pathways. Phthalate metabolites also show moderate clustering, such as between MEHHP and MECPP (0.66). Overall, these patterns highlight co-occurring chemical exposures and provide important context for understanding the interrelationships between multiple environmental exposure and depression.

### 3.4. Bayesian Kernel Machine Regression Analysis

#### 3.4.1. Posterior Inclusion Probability Analysis

Table 3 summarizes the Bayesian Kernel Machine Regression-derived group and conditional posterior inclusion probabilities (PIPs) for the environmental exposures evaluated in relation to depressive symptoms. The group PIP quantifies the overall importance of each chemical class in the mixture, whereas the conditional PIP reflects the relative contribution of individual constituents within their respective groups.

For the PFAS group (PFOA, PFOS), the group PIP is 0.3388, suggesting modest evidence that this class contributes to the mixture effect. Within the group, PFOA exhibits a higher conditional PIP (0.6297) than PFOS (0.3703), indicating that PFOA accounts for a larger share of the class-level signal. The metals group (lead, cadmium, mercury) has a group PIP of 0.3489; mercury shows the strongest conditional probability (0.3945), followed by lead (0.3100) and cadmium (0.2955), highlighting mercury’s relatively greater influence.

The organophosphate metabolites display the highest group PIP (0.7875), providing strong evidence that this class is important in the mixture. Diethylphosphate dominates within the group (conditional PIP 0.7211), whereas dimethylphosphate (0.1752) and dimethylthiophosphate (0.1037) contribute smaller individual effects. The phthalate biomarkers form a group with a PIP of 0.4391. MBP shows the highest conditional PIP (0.3696), while MEP, MEHHP, and MECPP contribute moderate signals, and MEHP exhibits the lowest probability (0.0916).

Taken together, the PIP profiles reveal considerable heterogeneity in the relative importance of exposures across and within chemical classes, with organophosphate metabolites emerging as the most influential group in their association with depressive symptoms.

#### 3.4.2. Univariate Exposure Response

**Figure 2:**
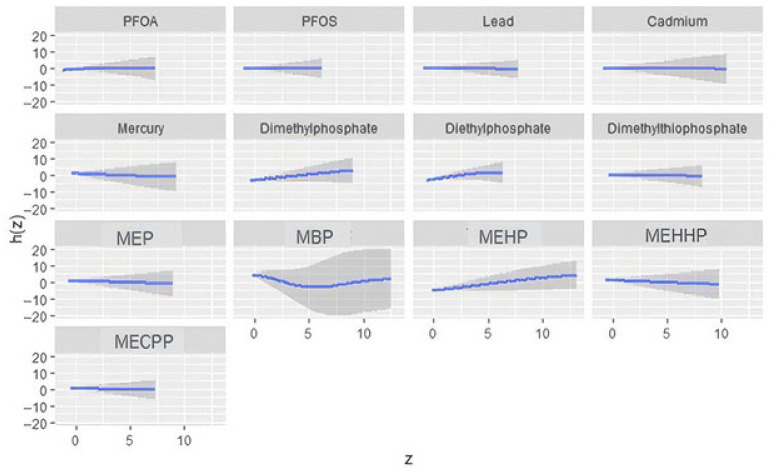
BKMR-estimated univariate exposure–response functions and corresponding 95% credible intervals for each environmental exposure, with all other components of the chemical mixture fixed at their median values. The blue curve represents the posterior mean estimate of the exposure–response function, and the shaded region indicates the 95% credible interval. Models were adjusted for alcohol use, smoking status, household income, education, age, sex, body mass index, and ethnicity.

Figure 2 presents the BKMR-estimated univariate exposure–response functions and their associated 95% credible intervals for PFAS (PFOA, PFOS), metals (lead, cadmium, mercury), organophosphate metabolites (dimethylphosphate, diethylphosphate, dimethylthiophosphate), and phthalate biomarkers in relation to depressive symptoms. These curves depict the posterior mean effect of each exposure while holding all other components of the mixture at their median values. The patterns reveal substantial heterogeneity across chemicals. Dimethylphosphate, MBP, and MEHP display marked nonlinear responses, suggesting potential threshold or plateau behavior. In contrast, mercury and PFOS show relatively flat trajectories with broader intervals, indicating weaker or less certain associations. Overall, the figure highlights that some exposures exhibit more pronounced univariate relationships with depressive symptoms, whereas others contribute minimal signal under the BKMR framework.

#### 3.4.3. Bivariate Exposure Response

**Figure 3:**
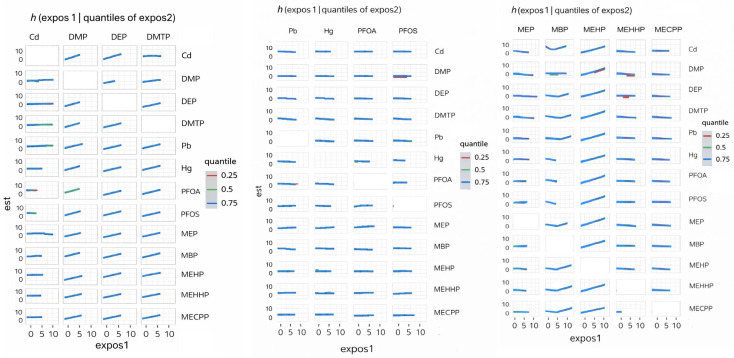
BKMR-estimated conditional bivariate exposure–response functions illustrating how the association between one exposure (Expos1) and depressive symptoms varies across quantiles (0.25, 0.50, and 0.75) of a second exposure (Expos2), with all remaining components of the chemical mixture fixed at their median values. Lines represent posterior mean estimates, and color denotes the quantile of Expos2. Models were adjusted for alcohol use, smoking status, household income, education, age, sex, body mass index, and ethnicity. Exposure abbreviations: Pb (lead), Cd (cadmium), Hg (mercury), DMP (dimethylphosphate), DEP (diethylphosphate), DMTP (dimethylthiophosphate), MEP (mono-ethyl phthalate), MBP (mono-n-butyl phthalate), MEHP (mono-(2-ethylhexyl) phthalate), MEHHP (mono-(2-ethyl-5-hydroxyhexyl) phthalate), and MECPP (mono-(2-ethyl-5-carboxypentyl) phthalate).

Figure 3 displays the BKMR-derived bivariate exposure–response functions, illustrating how the estimated effect of one exposure (expos1) on depressive symptoms changes across different levels of a second exposure (expos2). Expos2 is evaluated at the 0.25, 0.50, and 0.75 quantiles to represent low, medium, and high co-exposure conditions. The trajectories highlight whether the influence of expos1 varies as expos2 increases, providing insight into potential effect modification within the mixture.

#### 3.4.4. Overall Exposure Effect

**Figure 4:**
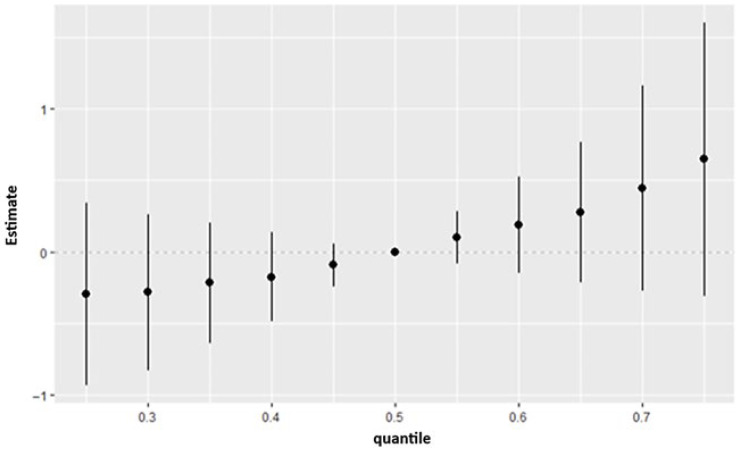
BKMR-estimated contrasts for each exposure evaluated across quantiles ranging from 0.25 to 0.75, with all effects expressed relative to the median (0.50 quantile). Points represent posterior mean estimates at each quantile, and vertical lines denote the corresponding 95% credible intervals.

Figure 4 summarizes the BKMR-estimated overall exposure effects on depressive symptoms across quantiles spanning 0.25 to 0.75. Each point represents the posterior mean contrast at a given quantile of the exposure distribution, and the vertical lines display the associated 95% credible intervals. The pattern of estimates indicates meaningful heterogeneity in the exposure–response relationship across the outcome distribution. At lower quantiles (0.25–0.45), the posterior means are predominantly negative, although the credible intervals consistently include zero, suggesting uncertainty. Around the median (0.50 quantile), the estimated effect is centered near zero, indicating minimal deviation from the null at the midpoint. In contrast, higher quantiles (0.55–0.75) exhibit increasingly positive posterior means, with larger effect sizes emerging as the quantile increases. Although uncertainty remains—reflected by intervals that still overlap zero—the upward trajectory suggests stronger positive associations between exposure and depressive symptoms among individuals in the higher portion of the outcome distribution.

#### 3.4.5. Single Exposure Effect Analysis

**Figure 5:**
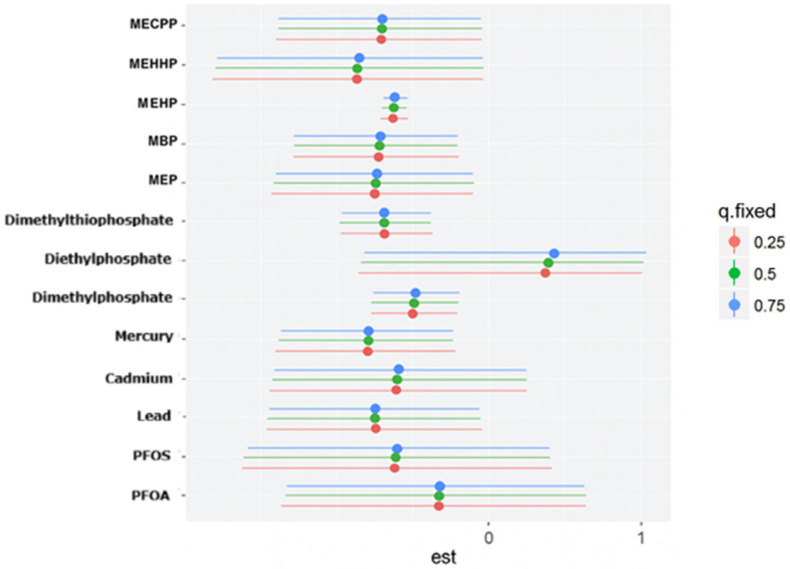
BKMR-estimated single-exposure effects of each environmental chemical on depressive symptoms, with all remaining exposures fixed at the 0.25 (red), 0.50 (green), or 0.75 (blue) quantiles. For each chemical, points denote posterior mean estimates and horizontal lines indicate the corresponding 95% credible intervals.

Figure 5 presents the BKMR-derived single-exposure functions for depressive symptoms, summarizing how the estimated response changes when an exposure is shifted from the 0.25 to the 0.75 quantile while the remaining components of the mixture are fixed at the 0.25, 0.50, or 0.75 quantile. Evaluated exposures include PFAS (PFOA, PFOS), metals (lead, cadmium, mercury), organophosphate metabolites (dimethylphosphate, diethylphosphate, dimethylthiophosphate), and phthalate biomarkers. For each chemical, the posterior mean effect under the three co-exposure settings is shown with color-coded points (blue for 0.25, green for 0.50, red for 0.75), and the accompanying horizontal lines denote the corresponding 95% credible intervals. This figure highlights how the estimated impact of a single exposure varies depending on the broader mixture context. Among the organophosphate metabolites, diethylphosphate shows the strongest positive single-exposure effect, though uncertainty remains evident from its wide credible interval.

**Figure 6:**
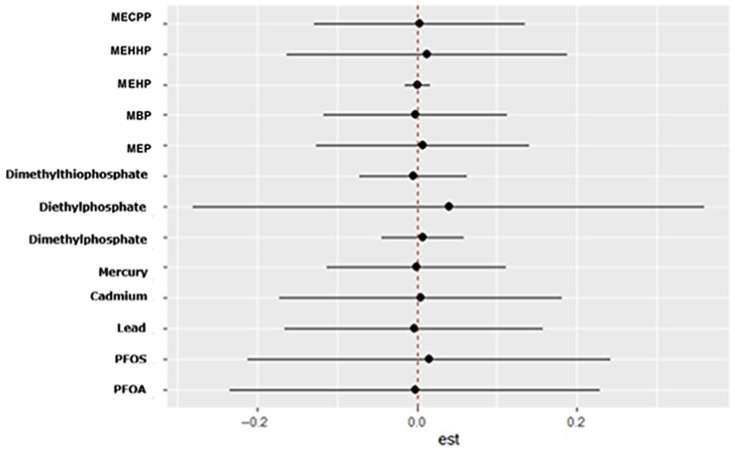
BKMR-estimated single-variable interaction effects for all environmental exposures. Each point and interval represents how the estimated effect of shifting an exposure from the 0.25 to the 0.75 quantile changes when all other exposures are held at either the 0.25 or the 0.75 quantile. Black dots denote posterior mean estimates, and horizontal lines show the corresponding 95% credible intervals.

Figure 6 displays the BKMR-estimated single-variable interaction functions for all environmental exposures. For each chemical, the posterior mean contrast is shown along the *x*-axis, with the associated 95% credible interval depicted by the horizontal line. The black points represent the posterior mean interaction estimates, and the vertical red reference line at zero indicates no departure from the null. These summaries characterize how the estimated effect of moving an exposure from the 0.25 to the 0.75 quantile differs when the remaining components of the mixture are fixed at either the lower or upper quantile. Among the exposures examined, diethylphosphate exhibits the most pronounced departure from the null, though the wide interval underscores substantial uncertainty in its interaction effect.

## 4. Discussion

This study explored the association between urinary phthalate and organophosphate metabolites and depressive symptoms in a U.S. sample. While most environmental exposures, including PFAS and heavy metals, did not show significant linear associations with depression, two metabolites, Dimethylphosphate and MEHP, emerged as significant predictors of higher PHQ-9 scores. The absence of strong associations for metals in this analysis may reflect relatively low exposure levels in the 2017–2018 NHANES cycle, limited variability, or competing mixture effects that attenuate individual metal signals when evaluated jointly. These findings highlight the importance of focusing on specific chemical exposures within complex mixtures, as not all compounds contribute equally to mental health outcomes.

The significant association between Dimethylphosphate and depressive symptoms is consistent with prior evidence linking organophosphate exposure to neurobehavioral disturbances. Organophosphates inhibit acetylcholinesterase, leading to excess acetylcholine accumulation and disrupted cholinergic signaling [32]. Chronic exposure has been associated with irritability, mood swings, and depression, particularly in agricultural workers and vulnerable populations [33]. The borderline association observed with Diethylphosphate further supports the possibility that organophosphate metabolites collectively influence depression risk, even if individual effects vary in strength.

These findings are further supported by recent population-based evidence from Zhao et al., [34] who reported robust positive associations between urinary dimethyl phosphate concentrations and both depression scores and depression severity among U.S. adults using NHANES 2015–2018 data. In that study, increasing levels of dimethyl phosphate were associated with higher PHQ-9 scores and substantially elevated odds of both mild and major depression, reinforcing the role of this metabolite as a key marker of organophosphate-related neurotoxicity. Taken together, the consistency of associations observed across independent analyses and analytic approaches strengthens the evidence that low-level, chronic exposure to organophosphate metabolites may contribute to depressive symptom burden in the general population.

The positive association with MEHP highlights the role of phthalates as endocrine disruptors. Phthalates interfere with hormone regulation, stress response pathways, and neurodevelopmental processes, all of which are critical to emotional well-being [35]. Although the effect size was small, the statistical significance indicates that even modest elevations in phthalate exposure may contribute to depressive symptoms. This finding aligns with earlier NHANES analyses reporting associations between urinary phthalates and adult depression [10], reinforcing the plausibility of endocrine disruption as a mechanism underlying mood disorders. The results of the linear regression should be taken with caution as it fails to capture non-linear effects hence the use of BKMR.

The BKMR analysis provided additional insights into mixture effects. Organophosphate metabolites demonstrated the highest group-level importance, suggesting that their collective influence on depression is stronger than that of PFAS, metals, or phthalates. Within this group, Diethylphosphate showed the highest conditional importance, while Dimethylphosphate contributed less individually. Phthalates exhibited moderate group importance, with variability across individual metabolites. The present findings align with prior epidemiologic evidence linking organophosphate exposure to adverse mental health outcomes while extending this literature through a mixture-based analytic framework. Harrison and Mackenzie Ross [36] reported elevated levels of self-reported anxiety and depressive symptoms among individuals with long-term, low-level occupational exposure to organophosphate pesticides. Their study relied on cumulative exposure assessment derived from occupational history and group-based comparisons. In contrast, the current analysis used biomarker-based exposure measurements and evaluated depressive symptoms on a continuous scale, allowing for detection of subclinical symptom burden in the general population. The identification of diethylphosphate as the most influential organophosphate metabolite within the mixture is consistent with earlier observations of mood-related effects associated with organophosphate exposure, while providing greater resolution regarding the relative contribution of specific metabolites. Moreover, the predominance of organophosphate metabolites over PFAS, metals, and phthalates in the mixture analysis underscores the importance of evaluating co-occurring environmental exposures to more accurately characterize their relative influence on depressive symptomatology. These results emphasize the need to consider environmental exposures as mixtures rather than isolated compounds, as interactive and non-linear effects may better explain observed health outcomes [37].

The bivariate and univariate exposure–response BKMR plots further suggest potential nonlinear or threshold effects for several metabolites, including DMP, MBP, and MEHP. Such patterns indicate that health risks may intensify once exposures exceed certain levels or when co-exposures interact synergistically. These nonlinearities may also explain why traditional regression models, which assume linearity, fail to detect associations for some exposures.

In the single-variable BKMR analysis, diethylphosphate exhibited the largest marginal effect on depressive symptoms across the interquartile range, indicating that variation in this metabolite contributed more strongly to the outcome than any other exposure evaluated. Diethylphosphate also suggested the strongest evidence of interaction, as its exposure–response relationship varied according to the background levels of the remaining chemicals in the mixture. These findings are directly consistent with the study by Wu et al. [38], who analyzed NHANES data using weighted quantile sum regression and Bayesian kernel machine regression and reported diethylphosphate as the highest-weighted contributor to depression risk within the organophosphate mixture, along with a significant positive overall mixture effect. While Wu et al. primarily emphasized the combined effect of organophosphate metabolites and their relative importance based on mixture weights, the present analysis extends their findings by demonstrating that the influence of diethylphosphate is strongly conditioned by co-exposure context. Specifically, the observed interaction patterns indicate that the effect of diethylphosphate on depressive symptoms is not isolated but is shaped by concurrent exposure profiles, which is consistent with established principles of mixture toxicology. Collectively, these results position diethylphosphate as a key driver within the organophosphate group and a major contributor to the mixture-associated increase in depressive symptoms detected in the BKMR model.

The overall exposure effect results showed that the combined mixture of PFAS, metals, phthalates, and organophosphate metabolites produced a consistent upward shift in depression risk across the higher quantiles of the outcome distribution. Although the credible intervals indicated some uncertainty, the pattern suggested that the joint burden of these chemicals may exert a stronger influence on depressive symptoms than any single exposure considered alone. This matches prior work which suggests that exposure to multiple pollutants including metals and PFAS cumulatively are associated with depression [2,39].

### 4.1. Limitations

Several limitations should be acknowledged. First, the cross-sectional design restricts causal inference. Second, self-reported depressive symptoms via the PHQ-9, while validated, may not fully capture clinical diagnoses of depression. Third, the study did not account for long-term exposure histories or potential interactions with genetic susceptibility, which may influence vulnerability. Future longitudinal studies with repeated biomarker measurements, clinical assessments, and mechanistic investigations are needed to strengthen causal interpretations and clarify dose–response relationships. The relatively modest analytic sample size reflects the requirement for complete biomarker data across multiple chemical classes, a common constraint in multi-exposure NHANES analyses, and may limit statistical power for detecting smaller effects.

### 4.2. Practical and Policy Implications

The findings of this study have several important implications for public health practice and environmental policy [40]. First, the identification of organophosphate metabolites as key contributors within the exposure mixture suggests that pesticide-related exposures may represent a modifiable environmental risk factor for depressive symptoms at the population level [41]. These results support continued efforts to reduce organophosphate exposure through regulatory oversight, improved occupational and residential exposure controls, and public education on pesticide use. Second, the observed mixture-related patterns underscore the limitations of single-chemical risk assessment approaches and highlight the need for cumulative and mixture-based frameworks in environmental health surveillance and policy decision-making [42]. Incorporating mixture-oriented evidence into regulatory evaluations may improve the identification of populations at risk and better reflect real-world exposure scenarios relevant to mental health outcomes.

### 4.3. Study Strengths

This study has several notable strengths. The simultaneous evaluation of multiple chemical classes reflects real-world exposure conditions and advances the literature beyond traditional single-exposure analyses. In addition, the application of Bayesian Kernel Machine Regression allowed for flexible modeling of non-linear and interactive exposure–response relationships, providing insights into mixture effects that would not be captured using conventional regression approaches. Finally, the integration of multiple analytic strategies strengthens confidence in the observed patterns and supports the robustness of the findings.

## 5. Conclusions

This study highlights the potential role of environmental toxicants, in shaping mental health outcomes. While most exposures examined did not show strong linear associations with depressive symptoms, Dimethylphosphate and MEHP emerged as significant predictors of higher PHQ-9 scores. Mixture modeling further revealed that organophosphates exert the greatest collective influence, emphasizing the importance of evaluating combined exposures rather than isolated compounds. By integrating traditional regression with Bayesian Kernel Machine Regression (BKMR), this research demonstrates the value of advanced statistical approaches in uncovering complex, non-linear, and interactive effects of chemical mixtures on depression. The findings contribute to a growing body of evidence linking environmental exposures to mood disorders and underscore the need for continued investigation into the biological mechanisms underlying these associations. Ultimately, this study reinforces the importance of public health strategies aimed at reducing exposure to endocrine-disrupting and neurotoxic chemicals. It also calls for future longitudinal research to clarify causal pathways and strengthen the evidence base for policy interventions that protect vulnerable populations from environmental risks to mental health.

## Figures and Tables

**Table 1:** (**a**) Descriptive statistics for categorical variables. (**b**) Descriptive statistics for critical variables of the study participants.

(a)			
Variable	Description	Frequency (n)	Percentage (%)
Sex		179	
	Male	109	60.89
	Female	70	39.11
Ethnicity	Mexican American	21	11.73
	Other Hispanic	8	4.46
	Non-Hispanic white	80	44.69
	Non-Hispanic Black	48	26.82
	Non-Hispanic Asian	8	4.47
	Other races, including multiracial	14	7.82
(**b**)			
**Variable**	**N**	**Mean**	**St. Dev**
PHQ	179	4.84	5.38
PFOA	179	1.60	1.24
PFOS	179	5.64	5.62
Lead	179	1.47	1.45
Cadmium	179	1.13	1.13
Mercury	179	1.14	2.48
Dimethylphosphate	179	3.00	7.70
Diethylphosphate	179	4.39	6.36
Dimethylthiophosphate	179	1.68	4.24
MEP	179	16.32	24.39
MBP	179	3.26	14.49
MEHP	179	150.35	970.81
MEHHP	179	8.27	11.45
MECPP	179	1.71	2.22
Age	179	44.39	14.91
BMI	179	29.57	8.35

Note PHQ = PHQ-9, a measure of depressive symptoms.

**Table 2:** Linear regression results for depression.

Variable	* Coef	Std. Err.	Pr(>|t|)	95% Conf. Interval
PFOA	0.032	0.3999	0.4294	−0.4731, 1.1067
PFOS	0.011	0.1066	0.9203	−0.2000, 0.2214
Lead	−0.074	0.3094	0.8120	−0.6849, 0.5375
Cadmium	0.021	0.4231	0.9612	−0.8151, 0.8564
Mercury	−0.050	0.2049	0.8084	−0.4545, 0.3549
Dimethylphosphate	0.154	0.0587	** 0.0098	0.0375, 0.2695
Diethylphosphate	0.126	0.0679	0.0648	−0.0079, 0.2606
Dimethylthiophosphate	−0.067	0.0994	0.5037	−0.2631, 0.1298
MEP	−0.008	0.0184	0.6753	−0.0442, 0.0287
MBP	−0.022	0.0340	0.5229	−0.0891, 0.0455
MEHP	0.001	0.0003	** 0.0043	0.0003, 0.0019
MEHHP	−0.018	0.0530	0.7408	−0.1224, 0.0872
MECPP	−0.124	0.2635	0.6391	−0.6443, 0.3966

* Adjusted for alcohol, smoking, income, education, age, sex, BMI and ethnicity. ** Statistically significant.

**Table 3:** BKMR Analysis of depressive symptoms: Group and Conditional Posterior Inclusion Probabilities.

Variable	Group	Group PIP	Conditional PIP
PFOA	1	0.3388	0.6297
PFOS	1	0.3388	0.3703
Lead	2	0.3489	0.3100
Cadmium	2	0.3489	0.2955
Mercury	2	0.3489	0.3945
Dimethylphosphate	3	0.7875	0.1752
Diethylphosphate	3	0.7875	0.7211
Dimethylthiophosphate	3	0.7875	0.1037
MEP	4	0.4391	0.1926
MBP	4	0.4391	0.3696
MEHP	4	0.4391	0.0916
MEHHP	4	0.4391	0.1985
MECPP	4	0.4391	0.1477

## Data Availability

The data presented in this study are openly available on the CDC NHANES site at https://wwwn.cdc.gov/nchs/nhanes/continuousnhanes/overview.aspx?BeginYear=2017 (accessed on 1 December 2025).

## References

[1] Liu J., Ning W., Zhang N., Zhu B., Mao Y. (2024). Estimation of the global disease burden of depression and anxiety between 1990 and 2044: An analysis of the global burden of disease study 2019. Healthcare.

[2] Ogundare O., Obeng-Gyasi E. (2025). The Combined Effects of Per-and Polyfluoroalkyl Substances, Metals, and Behavioral and Social Factors on Depressive Symptoms. Med. Sci..

[3] Tang C., Wang Y., Hong H. (2024). Unraveling the link between heavy metals, perfluoroalkyl substances and depression: Insights from epidemiological and bioinformatics strategies. Ecotoxicol. Environ. Saf..

[4] Nguyen H.D., Kim M.-S. (2023). Interactions between Cadmium, Lead, Mercury, and Arsenic and Depression: A Molecular Mechanism Involved. J. Affect. Disord..

[5] Li P., Zhang H., Han Y., Xie S., Li W., Kong L., Zhang Y. (2025). Phthalate metabolite mixtures and dose-response associations with depressive symptoms in US adults. Ecotoxicol. Environ. Saf..

[6] Wu Y., Song J., Zhang Q., Yan S., Sun X., Yi W., Pan R., Cheng J., Xu Z., Su H. (2023). Association between organophosphorus pesticide exposure and depression risk in adults: A cross-sectional study with NHANES data. Environ. Pollut..

[7] Wang C.-J., Yang H.-W., Li M.-C. (2023). Association between phthalate exposure and the risk of depressive symptoms in the adult population of the United States. Chemosphere.

[8] Kim M.R., Jung M.K., Jee H.M., Ha E.K., Lee S., Han M.Y., Yoo E.-G. (2024). The association between phthalate exposure and pubertal development. Eur. J. Pediatr..

[9] Wang Y., Qian H. (2021). Phthalates and their impacts on human health. Healthcare.

[10] Shiue I. (2015). Urinary heavy metals, phthalates and polyaromatic hydrocarbons independent of health events are associated with adult depression: USA NHANES, 2011–2012. Environ. Sci. Pollut. Res..

[11] Barrett J.R. (2005). Phthalates and baby boys: Potential disruption of human genital development. Environ. Health Perspect..

[12] Lottrup G., Andersson A.M., Leffers H., Mortensen G.K., Toppari J., Skakkebaek N., Main K.M. (2006). Possible impact of phthalates on infant reproductive health. Int. J. Androl..

[13] Hannon P.R., Flaws J.A. (2015). The effects of phthalates on the ovary. Front. Endocrinol..

[14] Antoniou E.E., Otter R. (2024). Phthalate exposure and neurotoxicity in children: A systematic review and meta-analysis. Int. J. Public Health.

[15] Yu Z.-X., Mo H.-Y., Shan C.-H., Zhao Y.-M., Zhou J.-X., Wang Y.-F., Liu Y., Tong J., Geng M.-L., Wu X. (2025). Risk of Neurodevelopmental Disorders in Preschool Children Associated with the Longitudinal Trajectory of Phthalates during Pregnancy: Potential Mechanisms Based on Metabonomics of Cord Blood. Environ. Sci. Technol..

[16] Yang Y., Wan S., Yu L., Liu W., Song J., Shi D., Zhang Y., Chen W., Qiu W., Wang B. (2025). Phthalates exposure, biological aging, and increased risks of insulin resistance, prediabetes, and diabetes in adults with metabolic dysfunction-associated steatotic liver disease. Diabetes Metab..

[17] Stojanoska M.M., Milosevic N., Milic N., Abenavoli L. (2017). The influence of phthalates and bisphenol A on the obesity development and glucose metabolism disorders. Endocrine.

[18] Dutta S., Haggerty D.K., Rappolee D.A., Ruden D.M. (2020). Phthalate exposure and long-term epigenomic consequences: A review. Front. Genet..

[19] Li J., Covaci A., Chen D. (2023). Environmental chemicals and adverse pregnancy outcomes: Placenta as a target and possible driver of pre-and postnatal effects. Crit. Rev. Environ. Sci. Technol..

[20] Uwaifo F., John-Ohimai F. (2020). Dangers of organophosphate pesticide exposure to human health. Matrix Sci. Medica.

[21] Silva V.B., Hellinger R., Orth E.S. (2020). Organophosphorus Compounds. Coastal and Deep Ocean Pollution.

[22] Seo S.-H., Batterman S., Karvonen-Gutierrez C.A., Park S.K. (2025). Determinants of urinary dialkyl phosphate metabolites in midlife women: The Study of Women’s Health Across the Nation Multi-Pollutant Study (SWAN-MPS). J. Expo. Sci. Environ. Epidemiol..

[23] Subedi B., Sullivan K.D., Dhungana B. (2017). Phthalate and Non-Phthalate Plasticizers in Indoor Dust from Childcare Facilities, Salons, and Homes across the USA. Environ. Pollut..

[24] Ragnarsdottir K.V. (2000). Environmental fate and toxicology of organophosphate pesticides. J. Geol. Soc..

[25] Muñoz-Quezada M.T., Lucero B.A., Barr D.B., Steenland K., Levy K., Ryan P.B., Iglesias V., Alvarado S., Concha C., Rojas E. (2013). Neurodevelopmental effects in children associated with exposure to organophosphate pesticides: A systematic review. Neurotoxicology.

[26] Eskenazi B., Bradman A., Castorina R. (1999). Exposures of children to organophosphate pesticides and their potential adverse health effects. Environ. Health Perspect..

[27] Davies R., Ahmed G., Freer T. (2000). Chronic exposure to organophosphates: Background and clinical picture. Adv. Psychiatr. Treat..

[28] Panieri E., Baralic K., Djukic-Cosic D., Buha Djordjevic A., Saso L. (2022). PFAS molecules: A major concern for the human health and the environment. Toxics.

[29] Yi W., Xuan L., Zakaly H.M., Markovic V., Miszczyk J., Guan H., Zhou P.-K., Huang R. (2023). Association between per-and polyfluoroalkyl substances (PFAS) and depression in US adults: A cross-sectional study of NHANES from 2005 to 2018. Environ. Res..

[30] Wang Y., Xu T., Zhang Y., He Y., Fang J., Xu Y., Jin L. (2024). Interaction between depression and non-essential heavy metals (Cd, Pb, and Hg) on metabolic diseases. J. Trace Elem. Med. Biol..

[31] Kroenke K., Spitzer R.L. (2002). The PHQ-9: A new depression diagnostic and severity measure. Psychiatr. Ann..

[32] Aroniadou-Anderjaska V., Figueiredo T.H., de Araujo Furtado M., Pidoplichko V.I., Braga M.F. (2023). Mechanisms of organophosphate toxicity and the role of acetylcholinesterase inhibition. Toxics.

[33] Cancino J., Soto K., Tapia J., Muñoz-Quezada M.T., Lucero B., Contreras C., Moreno J. (2023). Occupational exposure to pesticides and symptoms of depression in agricultural workers. A systematic review. Environ. Res..

[34] Zhao H., Kang X. (2024). Associations of Depression Score with Dialkyl Phosphate Metabolites in Urine: A Cross-Sectional Study. Brain Sci..

[35] Lucaccioni L., Trevisani V., Passini E., Righi B., Plessi C., Predieri B., Iughetti L. (2021). Perinatal exposure to phthalates: From endocrine to neurodevelopment effects. Int. J. Mol. Sci..

[36] Harrison V., Mackenzie Ross S. (2016). Anxiety and Depression Following Cumulative Low-Level Exposure to Organophosphate Pesticides. Environ. Res..

[37] Silins I., Högberg J. (2011). Combined toxic exposures and human health: Biomarkers of exposure and effect. Int. J. Environ. Res. Public Health.

[38] Stallones L., Beseler C.L. (2016). Assessing the connection between organophosphate pesticide poisoning and mental health: A comparison of neuropsychological symptoms from clinical observations, animal models, and epidemiological studies. Cortex.

[39] Ogundare O., Obeng-Gyasi E. (2024). Association of the Combined Effects of Metals Exposure and Behavioral Factors on Depressive Symptoms in Women. Toxics.

[40] Möhring N., Ingold K., Kudsk P., Martin-Laurent F., Niggli U., Siegrist M., Studer B., Walter A., Finger R. (2020). Pathways for advancing pesticide policies. Nat. Food.

[41] Kham-Ai P., Chaichan M., Sripo N., Pongchangyou K., Weiangkham D., Heaton K. (2026). Pesticide Exposure and the Risk of Depression, Anxiety, and Suicide: A Meta-Analysis. West. J. Nurs. Res..

[42] Savitz D.A., Hattersley A.M. (2023). Evaluating chemical mixtures in epidemiological studies to inform regulatory decisions. Environ. Health Perspect..

